# A Rare Case of New-Onset Ulcerative Colitis in a Nonagenarian

**DOI:** 10.7759/cureus.26203

**Published:** 2022-06-22

**Authors:** Emmanuel U Emeasoba, Cece E Ibeson, Sanchit Kundal, Stefanie Biondi, Ifeanyi Nwosu, Shmuel Golfeyz, Michael Kantrowitz, Dimitry Khodorskiy

**Affiliations:** 1 Internal Medicine, Maimonides Medical Center, New York, USA; 2 Emergency Medicine, New York University Langone Health, New York, USA; 3 Gastroenterology, Maimonides Medical Center, New York, USA

**Keywords:** biologic agents, histologic remission, acute flare, ulcerative colitis, -year-old

## Abstract

Ulcerative colitis (UC) is one of two major types of inflammatory bowel disease (IBD). It is defined as a chronic idiopathic inflammatory disease limited to the colorectal mucosal layer and characterized by relapsing and remitting episodes of inflammation. UC almost invariably involves the rectum and extends proximally in a continuous distribution to part or the entire colon. Development of disease after 75 years of age is uncommon, with new-onset over the age of 80 accounting only for 1% of all new diagnoses. We present a case of a new onset UC in a 90-year-old patient presenting with painless hematochezia.

## Introduction

In the United States, inflammatory bowel disease (IBD) is quite prevalent, with over 3.1 million afflicted [1]. Approximately 15,000 new cases of ulcerative colitis (UC) are diagnosed each year [1], with an incidence of 9-20 cases per 100,000 person-years [2]. The overall median age of onset is 33; however, there is a bimodal distribution. Peak incidence occurs in the second to third decades of life, with a second, smaller peak occurring in the sixth decade [3]. Up to 15% of newly diagnosed IBD patients in the United States are elderly [4]. While the incidence of UC in individuals over the age of 60 is slated to rise in the next ten years, as the proportion of elderly in the population increases, only 10% are in their seventh decade or older. Of these, 65% are aged between 60-70, while 25% are aged between 70-80, and only 10% are over 80 years. Therefore, only 1% of total newly diagnosed UC patients are in their ninth decade or older [5].

Our manuscript was accepted as an abstract to the New York Chapter, American College of Physicians (NYACP) 2020, and also presented as a poster presentation on the 29th of February 2020 at the 2020 NYACP Resident and Medical Student Forum.

## Case presentation

A 90-year-old woman with atrial fibrillation (on metoprolol tartrate, not on anticoagulation), hypertension (on losartan), and gastroesophageal reflux disease (on pantoprazole) presented to the emergency department complaining of two weeks of bloody stools. Bowel movements occurred three to five times daily, sometimes at night, mostly watery in consistency and not associated with fever, abdominal pain, or tenesmus. A physical exam was pertinent for tachycardia with irregularly irregular heart rhythm without any hemodynamic instability. The patient had large palpable hemorrhoids without evidence of active bleeding. Laboratory studies identified normocytic anemia along with elevated C-reactive protein, erythrocyte sedimentation rate, and fecal calprotectin respectively. A computer tomography scan revealed diffuse diverticulosis and pan-colitis, prompting the initiation of intravenous (IV) antibiotics (ciprofloxacin/metronidazole).

**Figure 1:**
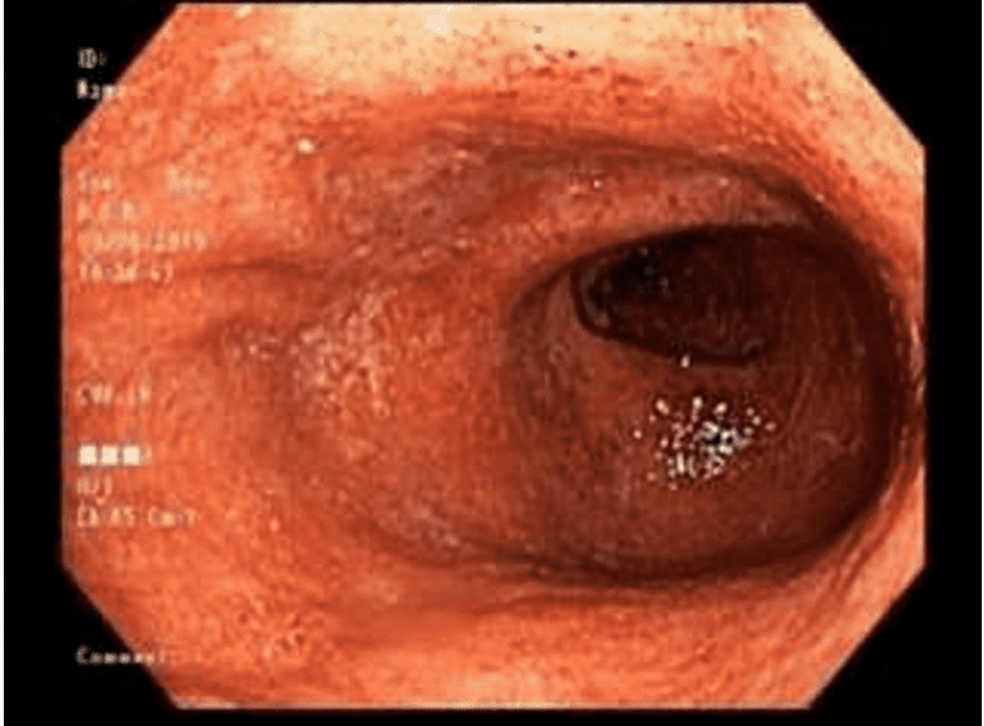
Rectum: erythematous, edematous and friable mucosa with loss of vascularity, and superficial ulcerations with exudate

**Figure 2:**
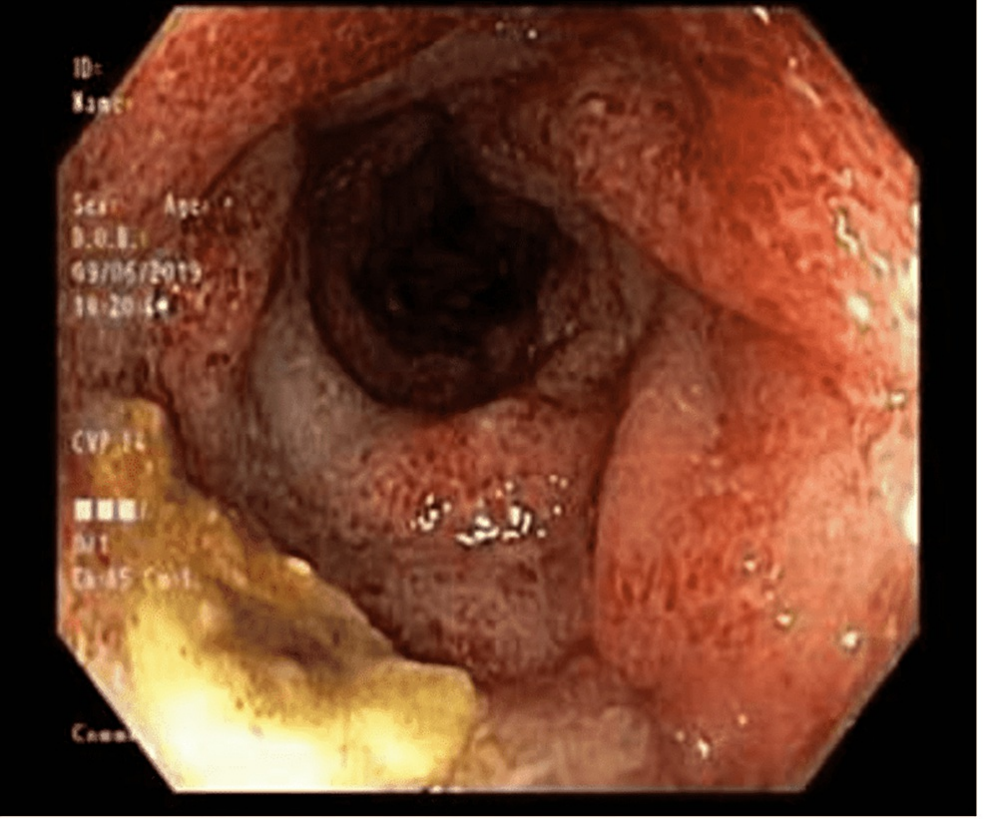
Sigmoid colon: erythematous, edematous and friable mucosa with loss of vascularity, and superficial ulcerations with exudate, as well as multiple small-mouthed diverticula

**Figure 3:**
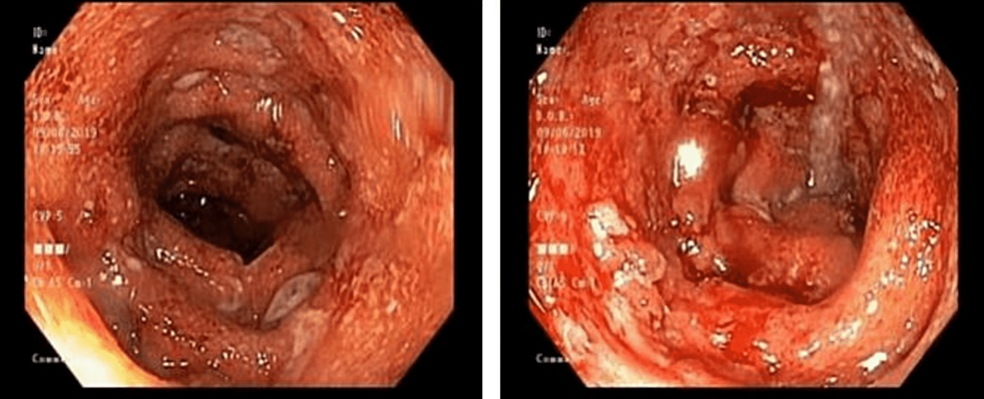
Descending colon: erythematous, edematous and friable mucosa with loss of vascularity, and superficial ulcerations with exudate, as well as multiple small-mouthed diverticula

The patient underwent a sigmoidoscopy, which revealed an erythematous, edematous, and friable mucosa with loss of vascularity and superficial ulcerations with exudate that extended from rectum to splenic flexure in a continuous manner, as well as severe diverticulosis and large internal hemorrhoids. Stool studies, including cultures, ova and parasites, and Clostridium difficile (C. diff) toxin A and B with antigen stool assays, were negative. Histopathologic evaluation of mucosal biopsies identified active colitis, cryptitis, and focal crypt abscesses with architectural distortion without evidence of viral inclusions, granulomas, or dysplasia, suggestive of ulcerative colitis, as shown in Figures 1-3.

A decision was made to start the patient on IV corticosteroids to induce remission, and the patient was subsequently placed on hydrocortisone due to a hospital shortage of methylprednisolone.

Anti-saccharomyces cerevisiae antibodies (ASCA) and perinuclear anti-neutrophilic cytoplasmic Abs (p-ANCA; also known as myeloperoxidase [MPO-ANCA]) were obtained and were found to be negative. In anticipation of an escalation of therapy, the QuantiFERON®-TB Gold test (QFGT), thiopurine methyltransferase activity level, and hepatitis B (Hep B) panel were sent. Results revealed positive hepatitis B surface antibody (HBsAb), negative hepatitis B surface antigen (HBsAg), positive hepatitis B core antibody, IgG (Hep Bc IgG), and negative hepatitis B core antibody, IgM (Hep Bc IgM), consistent with prior exposure with current immunity. As QFGT was indeterminate, purified protein derivative was placed and was found to be negative.

After three days of IV hydrocortisone, the number of bowel movements gradually decreased, hematochezia resolved, and she was discharged home on a prednisone taper regimen and oral mesalamine. The patient was educated on the need for indefinite hepatitis B therapy should the need for a biological agent for her treatment arise in the future.

## Discussion

In 95% of cases, UC involves the rectum, progresses in a continuous circumferential manner, and may involve the entire colon. While hematochezia is the most common clinical feature, it accounts for only 4-9% of all causes of lower gastrointestinal bleeding, with diverticulosis, colon cancer, and ischemic colitis accounting for 42-66%. Most patients with new-onset UC present with a combination of hematochezia, accompanied by dyschezia, diarrhea, abdominal pain, or tenesmus, but can present as an aggressive first flare. Elderly patients, in comparison to young patients with the new-onset disease, usually have minimal symptoms [6], and they more commonly present with isolated colonic inflammation and perianal fistulas [4]. The majority of elderly patients present with proctitis, proctosigmoiditis, or left-sided colitis [7]. Although hematochezia remains the most frequently described clinical finding in the elderly, it is commonly presumed to be secondary to more common causes, such as diverticular hemorrhage, bleeding vascular ectasia, colorectal malignancy, various colitides, and hemorrhoidal blood loss.

In this patient, lack of tenesmus and abdominal pain led to an initial diagnosis of hemorrhoidal bleeding. Moreover, findings of pancolitis and diverticulosis further clouded the diagnosis. Although no risk factors for infectious colitis were elicited, such as recent antibiotic use, travel history, or consumption of unusual or undercooked food, community-acquired C. diff infection has been reported [8]. Additionally, the patient was on long-term proton pump inhibitor therapy, making the diagnosis of microscopic colitis a possibility. While typically not associated with hematochezia, frequent diarrhea from the aforementioned colitidies could have led to irritation of large hemorrhoids [9]. Finally, considering known underlying atrial fibrillation, ischemic colitis remained high on the differential [10]. All of the abovementioned conditions were further assessed in several studies as confounding factors in diagnosing UC. They were shown to cause the rate of misdiagnosis at initial presentation in people over the age of 60 to as high as 60%, compared to 15% in patients younger than 60 [5]. Furthermore, patients with isolated disease confined to the distal colon and/or rectum have a 20.8% chance of being correctly diagnosed, as compared to 17.9% in those with extensive colonic involvement [7].

In addition to clinical assessment, diagnosing UC involves laboratory findings and endoscopic examination. Fecal calprotectin is sensitive to IBD. However, it is nonspecific and can also be elevated in infectious colitis and colon malignancies [11]. p-ANCA is positive in 60-70% of patients with UC but has low sensitivity. At the same time, ASCA is a non-sensitive indicator of Crohn's disease and is only rarely positive in UC patients [8]. Other laboratory findings are even less specific or sensitive.

About 54% of elderly patients newly diagnosed with UC require systemic corticosteroids due to disease severity [12]. According to Truelove and Witts criteria [13] and the Mayo Scoring System for Assessment of UC Activity [14], the patient had moderate to severe disease at presentation. After the resolution of an acute flare, the next step is an evaluation to determine whether an escalation of therapy, such as initiation of a biologic agent, is necessary for the maintenance of remission [15]. Although mucosal healing has been the gold standard in IBD treatment, with the development of biological agents, histologic remission is the new ultimate therapeutic goal, which is defined as the absence of inflammation or structural changes on mucosal biopsies.

## Conclusions

Although rare, new-onset UC could present in the elderly as an aggressive first flare. The patient had moderate to severe disease at presentation with an excellent response to IV corticosteroids. This case highlights the importance of recognizing UC as one of the differential diagnoses in elderly patients presenting with hematochezia. Prompt recognition could result in timely initiation of appropriate therapy, leading to disease remission and avoidance of life-threatening complications.
